# Caffeine in liver diseases: Pharmacology and toxicology

**DOI:** 10.3389/fphar.2022.1030173

**Published:** 2022-10-17

**Authors:** Liang Shan, Fengling Wang, Dandan Zhai, Xiangyun Meng, Jianjun Liu, Xiongwen Lv

**Affiliations:** ^1^ Department of Pharmacy, The Second People’s Hospital of Hefei, Hefei Hospital Affiliated to Anhui Medical University, Hefei, Anhui, China; ^2^ Anhui Province Key Laboratory of Major Autoimmune Diseases, Anhui Medical University, Hefei, Anhui, China; ^3^ Inflammation and Immune Mediated Diseases Laboratory of Anhui Province, Hefei, Anhui, China; ^4^ The Key Laboratory of Major Autoimmune Diseases, Hefei, Anhui, China

**Keywords:** caffeine, liver diseases, pharmacology, toxicology, adenosine, adenosine receptor

## Abstract

We have previously shown that adenosine A1AR antagonists, adenosine A2aAR antagonists, and caffeine have significant inhibitory effects on the activation and proliferation of hepatic stellate cells in alcoholic liver fibrosis. Many recent studies have found that moderate coffee consumption is beneficial for various liver diseases. The main active ingredient of coffee is caffeine, which is a natural non-selective adenosine receptor antagonist. Moreover, numerous preclinical epidemiological studies and clinical trials have examined the association between frequent coffee consumption and the risk of developing different liver diseases. In this review, we summarize and analyze the prophylactic and therapeutic effects of caffeine on various liver diseases, with an emphasis on cellular assays, animal experiments, and clinical trials. To review the prevention and treatment effects of caffeine on different liver diseases, we searched all literature before 19 July 2022, using “caffeine” and “liver disease” as keywords from the PubMed and ScienceDirect databases. We found that moderate coffee consumption has beneficial effects on various liver diseases, possibly by inhibiting adenosine binding to its receptors. Caffeine is a potential drug for the prevention and treatment of various liver diseases.

## 1 Introduction

Caffeine, one of the most widely consumed pharmacologically active ingredients in the world, is the main component of coffee and tea (Rawat et al., 2018; Alehaideb et al., 2021). One gram of Nescafe contains approximately 35 mg of caffeine, while 1 g of tea contains approximately 20–35 mg of caffeine. Most western countries, such as the United States, Britain, and France, have a greater tendency to drink coffee, while most eastern countries, such as China, India, and Japan, tend to favor tea (Alshabi et al., 2021). Scientists have paid increased attention to the relationship between caffeine and health because it is closely related to people’s daily lives (Leung et al., 2011). Traditionally, caffeine has been described as a potential drug of abuse, and it is widely stated that consuming too many caffeinated drinks is unhealthy (Barcelos et al., 2014). However, in recent years, an increasing number of interesting and controversial studies have stated that drinking caffeine can reduce the risk of various liver diseases, especially in alcoholics. Further research suggests that drinking more than two cups of coffee or tea per day significantly reduces the risk of chronic liver diseases in people at high risk of alcoholism, overweight, and diabetes (Higdon and Frei, 2006; Duseja, 2012).

**FIGURE 1:**
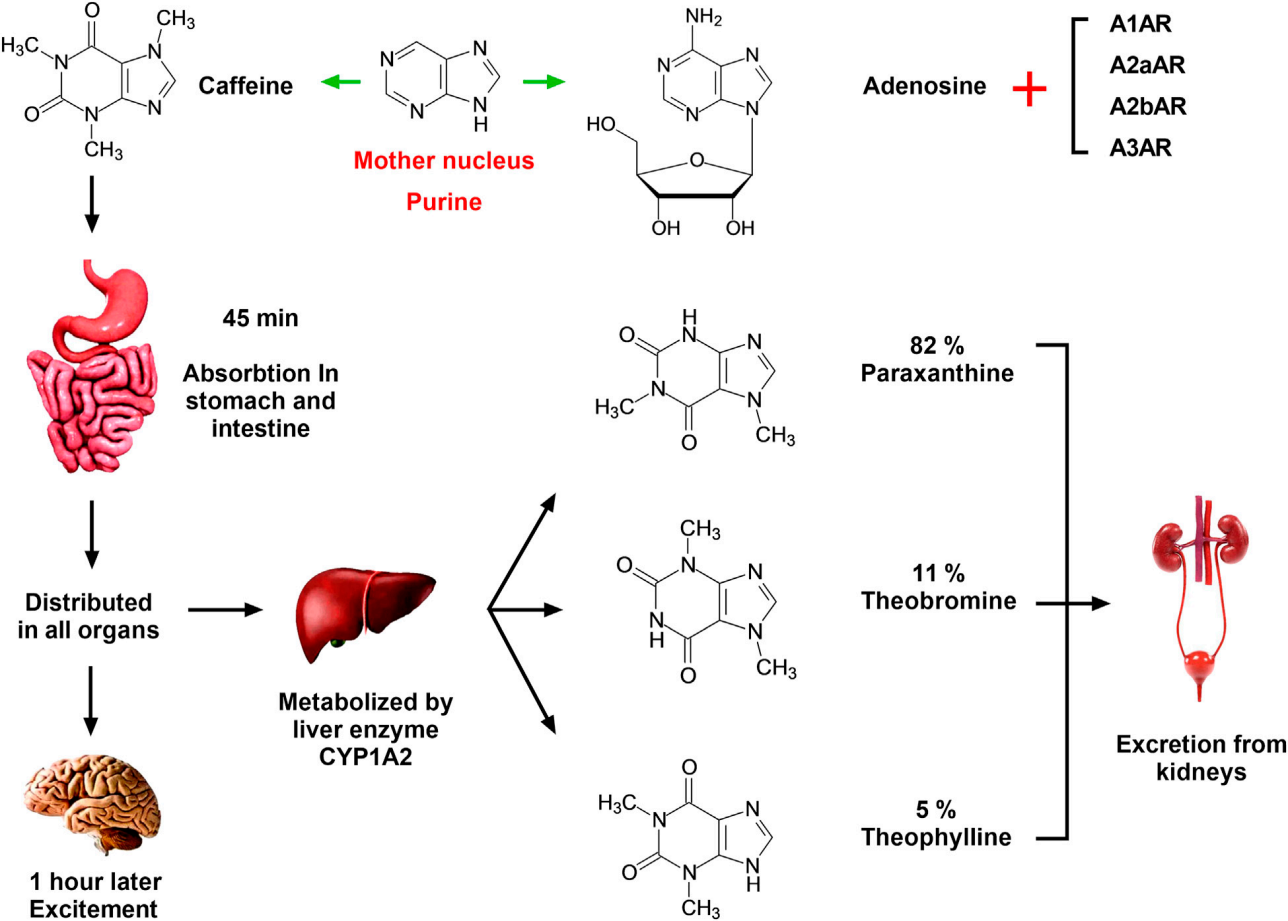
Absorption, distribution, metabolism, and excretion of caffeine in the human body. Caffeine has a similar chemical structure to adenosine, and the mother nucleus of both is purine. Therefore, caffeine is a natural non-selective antagonist of adenosine, which can competitively inhibit the binding of adenosine and ARs *in vivo* and play different physiological functions. Caffeine is completely absorbed by the stomach and small intestine within 45 min of oral administration, following which it is distributed in all organs of the body and enters the central nervous system through the blood within 1 h. In the central nervous system, caffeine can combine with ARs and cause central nervous system excitement. Caffeine is catalyzed by CYP1A2 in the liver to produce three primary metabolites: parxanthine (82%), theobromine (11%), and theophylline (5%). The conversion process is a first-order chemical kinetic reaction, and these compounds are further metabolized and eventually excreted in the urine.

The chemical structure of caffeine contains a purine ring, which chemically resembles adenosine and is a natural non-selective receptor antagonist of adenosine (Figure 1) (Eltzschig et al., 2012). Previous studies by our research team have shown that caffeine has a certain preventive effect on alcoholic liver fibrosis in rats, in which the cAMP-PKA-CREB signaling pathway is thought to play a role (Wang et al., 2015; Yang et al., 2015). We have previously used acetaldehyde to stimulate Hepatic Stellate cell-T6 (HSC-T6) cells in rats to establish an *in vitro* model of alcoholic liver fibrosis, and the results implicated the cAMP/PKA/SRC/ERK1/2/P38 MAPK signaling pathway as playing a key role, confirming the involvement of the adenosine signaling pathway in alcoholic liver fibrosis (Wang et al., 2014; Wang et al., 2015; Yang et al., 2015).

Recently, there are increasing reports that regular oral coffee is associated with reduced risk of various liver diseases (Saab et al., 2014). Therefore, to update the data in this field, we developed a research synthesis that includes the pathophysiological mechanisms, results of experimental and clinical studies, mechanisms of drug resistance and ways to overcome them, adverse effects of caffeine, recent patents in this field, and future developments. We conducted a small review of articles published before 19 July 2022 in PubMed and Web of Science, which were searched using the keywords “caffeine” and “liver disease.” As a result, we found caffeine to be a potential drug for preventing and treating various liver diseases.

## 2 Adenosine signaling in the liver

**FIGURE 2:**
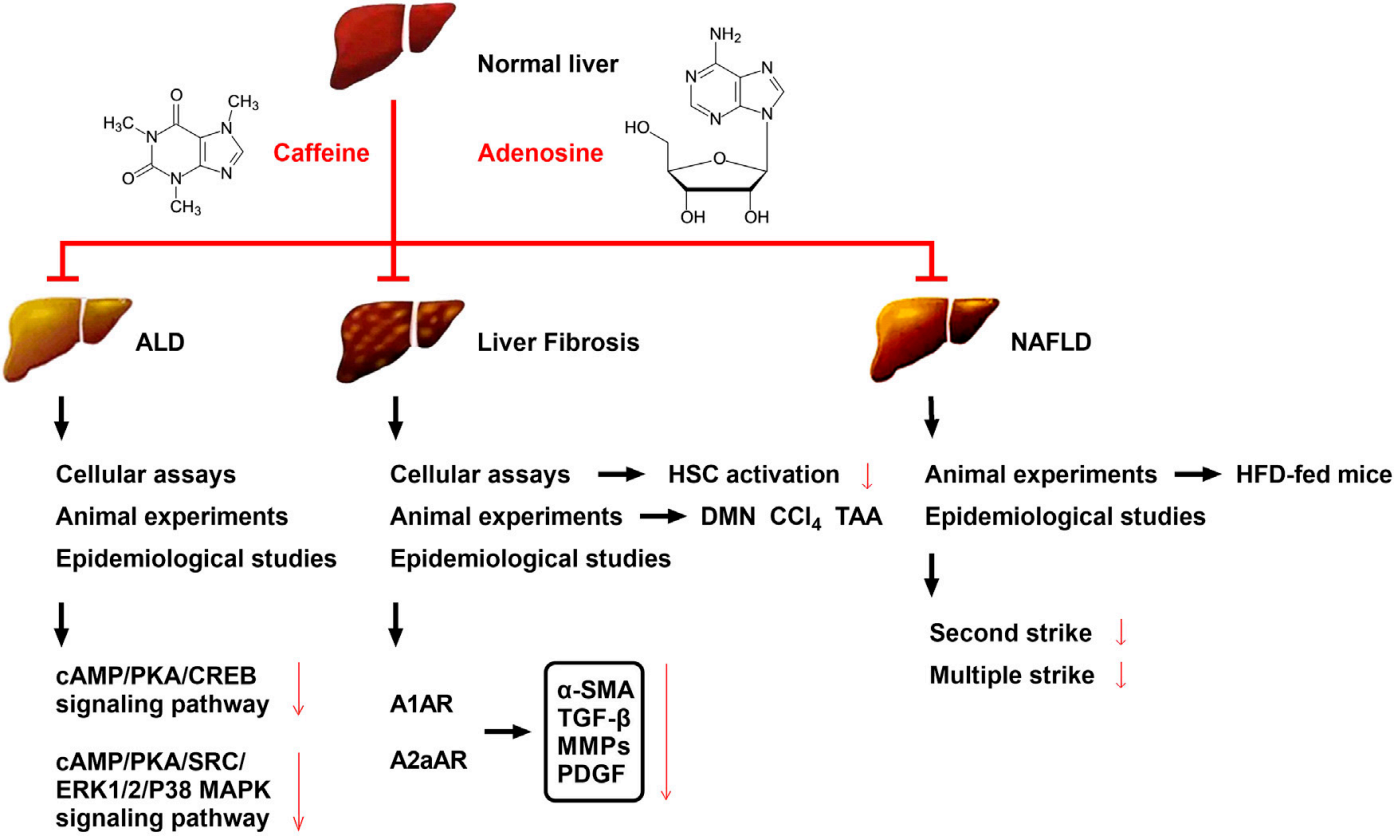
Effects and possible mechanisms of caffeine on reducing ALD, liver fibrosis, and NAFLD. The mechanism by which caffeine attenuates ALD may be related to its inhibition of the cAMP/PKA/CREB and cAMP/PKA/SRC/ERK1/2/P38 MAPK signaling pathways. The mechanism by which caffeine attenuates liver fibrosis may be related to its competitive inhibition of adenosine and AR binding (mainly A1AR, A2aAR, and A2bAR) in HSCs, which serves to reduce the levels of the extracellular fibrotic cytokines α-SMA, TGF-β, MMPs, and PDGF. Caffeine has been shown to reduce chemical toxicant-induced liver fibrosis in three animal models of liver fibrosis (DMN, CCl_4_, and TAA), and its effect on liver fibrosis has been further confirmed by multiple clinical trials. Caffeine attenuates HFD-induced NAFLD in mice, which has also been confirmed in several clinical trials; its mechanism may be related to the reduction of liver damage caused by the second strike and/or multiple strike.

**FIGURE 3:**
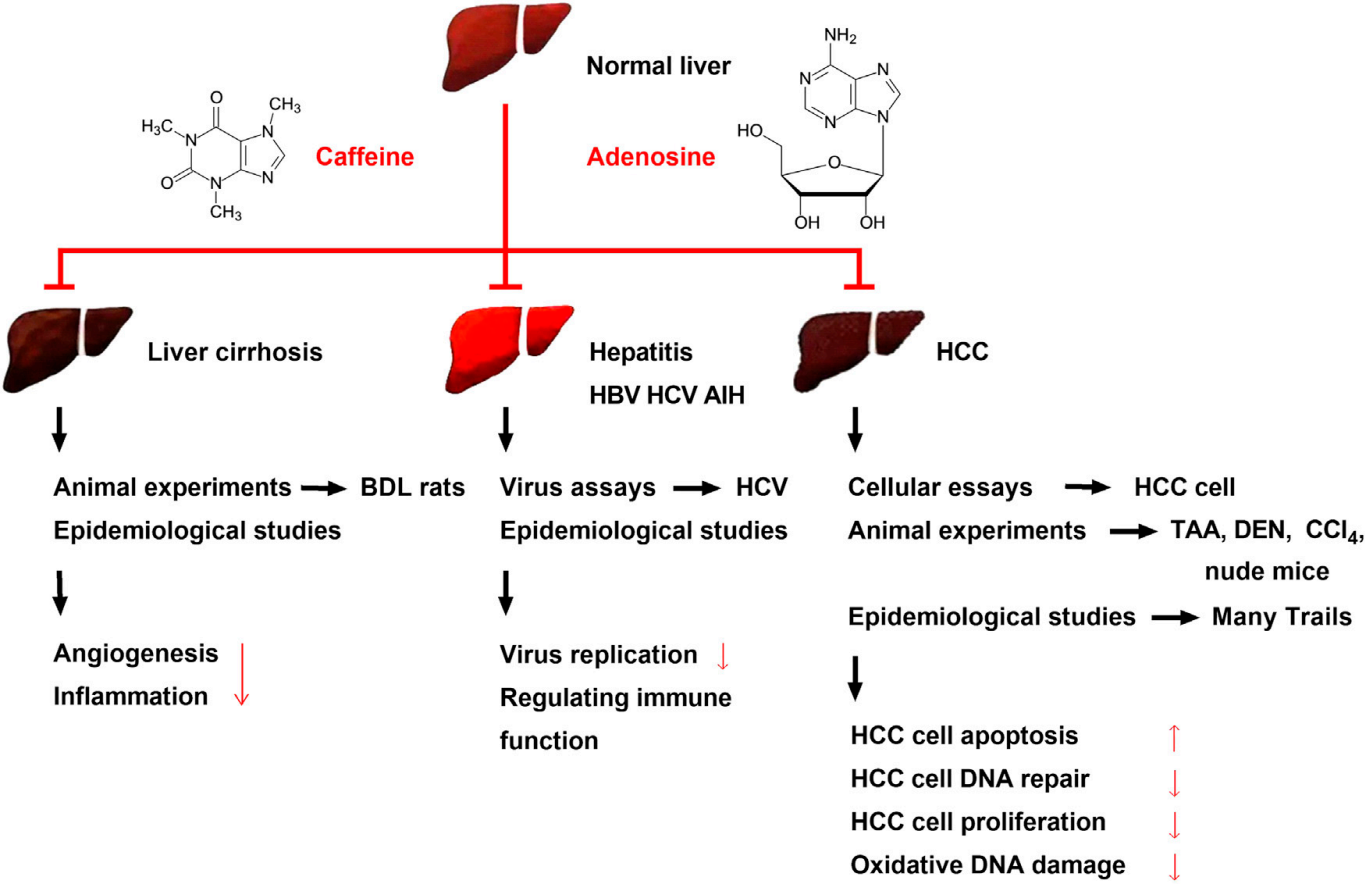
Effects of caffeine on alleviating liver cirrhosis, different types of hepatitis, and HCC and its possible mechanisms. Caffeine attenuates BDL-induced liver cirrhosis in rats. Several clinical trials have shown the same result, and the mechanism may be related to the ability of caffeine to inhibit inflammation and angiogenesis. Viral assays have found that caffeine can inhibit HBV and HCV, while clinical trials have found that caffeine can alleviate hepatitis B, C, and AIH; its mechanism may be related to the inhibition of hepatitis virus replication and regulation of immune function. Recent preclinical studies and multiple clinical trials have found that caffeine attenuates HCC. Caffeine inhibits the proliferation of various HCC cells and attenuates chemical toxicant (TAA, DEN, and CCl_4_)-induced HCC and xenograft HCC in nude mice. Large clinical trials initiated in several countries have found that the risk of HCC is reduced in people with an oral coffee consumption habit; this may be due to the ability of caffeine to inhibit HCC cell proliferation and oxidative DNA damage and reduce HCC cell DNA repair.

Caffeine is structurally similar to adenosine and is a non-selective adenosine receptor (AR) inhibitor that competently inhibits adenosine both *in vitro* and *in vivo* (van Dam et al., 2020). The release of adenosine triphosphate (ATP) from various cells in the liver (hepatocytes, Kupfer cells, and HSCs) increases when stimulated by various factors, including alcohol, acetaldehyde, chemical toxins, and radiation (Eltzschig et al., 2012; van Dam et al., 2020). When ATP is released extracellularly from stressed or damaged cells, it is rapidly hydrolyzed to adenosine monophosphate (AMP) and phosphoric acid by CD39/ENTPD1 expressed on the cell surface, releasing energy simultaneously, before being further hydrolyzed to adenosine by Ecto-5′-nucleotidase (NT5E, CD73). Adenosine binds to ARs to exert various physiological effects, while adenosine deaminase (ADA) plays a negative regulatory role, which hydrolyzes and inactivates adenosine. ARs have four subtypes, namely, A1AR, A2aAR, A2bAR, and A3AR, which are widely distributed in the body and play different physiological roles after binding with adenosine. Thus, the cascade of extracellular enzyme CD39-CD73-ADA co-regulates and stabilizes the adenosine level (Eltzschig et al., 2012; van Dam et al., 2020). We have previously shown that adenosine A1AR and A2AR of HSCs promote the activation and proliferation of HSCs after activation under the action of acetaldehyde; however, A1AR and A2AR have opposite regulatory effects on the cAMP-PKA-CREB signaling pathway. Additionally, adenosine A1AR antagonists, adenosine A2AR antagonists, and caffeine have significant inhibitory effects on the activation and proliferation of HSCs in alcoholic liver fibrosis (Wang et al., 2015; Yang et al., 2015). An overview of the relationship between the adenosine signaling pathway and various liver diseases is shown in Figures 2, 3.

### 2.1 Caffeine in alcoholic liver disease

Alcoholic liver disease (ALD) occurs as a result of the interaction of many factors, including oxidative stress, intestinal endotoxin, inflammatory mediators, and nutritional imbalance, which are directly or indirectly induced by the metabolism of ethanol and its derivatives (Li et al., 2021). Additionally, acetaldehyde, a metabolite of ethanol, has strong immunogenicity with the acetaldehyde adduct formed by various proteins, which can stimulate the body to produce antibodies and cause immune damage, leading to damage of important proteins, including protease and DNA (Li et al., 2021). Epidemiological studies have shown that coffee consumption can slow the development of chronic liver disease, including alcoholic cirrhosis (van Dam et al., 2020). Wang et al. established an alcoholic liver fibrosis rat model by intragastric administration of ethanol, followed by treatment with varying concentrations of caffeine (5, 10, and 20 mg/kg/day). The results showed that after 8–12 weeks of treatment, the levels of serum alanine aminotransferase (ALT), aspartate transaminase (AST), hyaluronic acid (HA), laminin (LN), N-terminal peptide of type III procollagen (PIIINP), and type IV collagen in the high-dose group were significantly reduced. Additionally, in an *in vitro* culture model of primary rat HSCs induced by acetaldehyde, it was confirmed that caffeine inhibits acetaldehyde-induced activation and proliferation of HSCs through the A2AR-mediated cAMP/PKA/CREB signaling pathway (Eltzschig et al., 2012; Wang et al., 2015). In conclusion, previous studies, both *in vivo* and *in vitro*, all support the conclusion that caffeine alleviates ALD (Figure 2).

### 2.2 Caffeine in liver fibrosis

Liver fibrosis is a pathophysiological process that refers to the abnormal proliferation of connective tissues in the liver induced by various pathogenic factors, including viral hepatitis, alcohol, fatty liver, and autoimmune diseases (Arauz et al., 2014; Ebadi et al., 2021). However, any liver injury also involves a process of liver fibrosis during repair and healing (Eraky et al., 2018; Romualdo et al., 2020). The beneficial effects of caffeine and coffee extracts on liver fibrosis have been demonstrated in several standard rodent models of experimental liver fibrosis induced by dimethylnitrosamine (DMN), carbon tetrachloride (CCl_4_), and thioacetamide (TAA), and protective effects of filtered coffee were found in most of the published studies (Furtado et al., 2012; Arauz et al., 2014; Eraky et al., 2018). Recently, researchers have found that caffeine can prevent CCl_4_-induced hepatic damage in mice through its antioxidant capacities (Pan et al., 2018). In contrast, one study showed that the intake of unfiltered Turkish coffee not only had no protective effect on CCl_4_-induced liver fibrosis but also significantly increased AST/ALT levels, thereby exacerbating CCl_4_-induced hepatotoxicity (Dranoff, 2018). As a caveat, given that the underlying mechanisms responsible for these differences have not been studied, more accurate animal studies are needed to confirm them.

Persistent inflammatory factors stimulate HSCs in the liver to secrete fibrotic factors that increase extracellular matrix (ECM) formation and ultimately lead to liver fibrosis (Eraky et al., 2018). In three rat models of CCl_4_, DMN, and TAA-induced hepatic fibrosis, coffee and caffeine intake reduced transforming growth factor-β (TGF-β) levels and thus inhibited HSC activation and proliferation (Arauz et al., 2014). During the progress of hepatic fibrosis, HSCs differentiated into myofibroblasts and promoted ECM secretion, a process commonly referred to as HSC activation. Caffeine intake can reduce the total content of hepatic collagen in rodent models of hepatic fibrosis. Activated HSCs also secrete matrix metalloproteinases (MMPs), whose activity is important in maintaining a balance between tissue repair and scar formation in hepatic fibrosis. Caffeine can significantly reduce the total MMP secretion in the liver. Besides, the expression of α-smooth muscle actin (α-SMA) is often a marker of HSC activation in hepatic fibrosis. Caffeine can decrease α-SMA expression in the whole liver, which may indicate decreased HSC activation and slow disease progression. In summary, *in vivo* studies have shown that the anti-fibrosis properties of caffeine converge with a reduction in HSC activation and proliferation (Amer et al., 2017).

HSCs are deemed the main effector cells in liver fibrosis (Eraky et al., 2018). Interestingly, human HSCs express all four ARs (A1AR, A2aAR, A2bAR, and A3AR), while mouse HSCs express all receptors, except A3AR. A2aAR, which regulates the function of HSCs, has been most widely studied (Yang et al., 2015). Indeed, following activation by extracellular adenosine, A2aAR on HSCs significantly upregulates collagen and TGF-β secretion, decreases MMP expression, and prevents the chemotaxis of HSCs to platelet-derived growth factor (PDGF). Additionally, liver inflammation activates HSCs, and the effect of PGE2 on HSC activation is changed from facilitatory to inhibitory when combined with caffeine, suggesting that caffeine effectively suppresses liver fibrosis during inflammation (Yamaguchi et al., 2021). These results suggest that the inhibition of the prefibrotic adenosine signal in HSCs is the mechanism underlying the anti-liver fibrosis effects of caffeine. The main possible mechanisms of the anti-liver fibrosis effects of caffeine are shown in Figure 2.

### 2.3 Caffeine in non-alcoholic fatty liver disease

Non-alcoholic fatty liver disease (NAFLD) refers to a clinicopathological syndrome characterized by hepatic steatosis, which is caused by other than alcohol and other unclear liver damage factors. The disease spectrum of NAFLD includes non-alcoholic fatty liver, also known as simple fatty liver, non-alcoholic fatty hepatitis, fatty liver fibrosis, cirrhosis, and hepatocellular carcinoma (HCC) (Fang et al., 2019; Mansour et al., 2021). CYP1A2 is an important enzyme in the liver, which catalyzes caffeine to form paraxanthine (82%), theobromine (11%), and theophylline (5%) (Perera et al., 2013; Garduno and Wu, 2021). Apparent caffeine clearance is considered a gold standard measurement of CYP1A2 activity. Previous studies have found that decreased CYP1A2 activity is associated with NAFLD progression, although more research is needed (Perera et al., 2013; Garduno and Wu, 2021). A study in 2019 showed that the prevalence rate of NAFLD in China was approximately 29.2%, with an increase of 14% in the past decade. However, the specific pathogenesis of NAFLD remains to be explored. The doctrine of the “multiple strike” theory, based on the “second strike” theory, is currently widely accepted, and includes lipotoxicity, mitochondrial dysfunction, endoplasmic reticulum stress, adipose tissue dysfunction, inflammatory cytokines, and enteric endotoxin multiple strike factors. Despite advances in research, there remain limited effective therapeutic drugs for NAFLD in clinical practice; thus, it is crucial to fully determine the pathogenesis of NAFLD to establish effective therapeutic targets (Sewter et al., 2021).

Many preclinical studies have shown that caffeine intake alleviates NAFLD and can even prevent NAFLD in high-fat-diet (HFD)-fed mice (Fang et al., 2019; Velázquez et al., 2020). Other studies have shown that Pu-erh tea extract (PTE) protected HFD-fed mice from developing NAFLD, whereas decaffeinated PTE showed no such anti-NAFLD effects (Helal et al., 2018; Fang et al., 2019). Additionally, further studies found that caffeine-stimulated muscle IL-6 mediates the alleviation of NAFLD. Researchers have found that consuming caffeine at a concentration of 0.5 mg/ml in drinking water for 16 weeks has a strong protective effect on HFD-induced NAFLD without affecting HFD-induced oxidative stress. The same method was used to feed IL-6 knockout and wild-type mice, with comparable levels of hepatic steatosis in HFD-fed IL-6^−/−^ and HFD-fed WT mice. The results showed that caffeine consistently protected against HFD-induced NAFLD in the wild-type mice, while the protective effects were abrogated in IL-6^−/−^ mice. Moreover, caffeine-induced STAT3 phosphorylation in the liver was inhibited in HFD-fed IL-6^−/−^ mice, and caffeine treatment reduced the ALT and AST levels in the HFD-fed control mice but not in the IL-6^−/−^ mice after 12 weeks. These results suggest that IL-6 is necessary for the protective effect of caffeine on NAFLD in mice (Fang et al., 2019).

Caffeine has shown inhibitory effects in most of the preclinical studies on the effects of caffeine on NAFLD/non-alcoholic steatohepatitis (NASH) (Dungubat et al., 2020; Velázquez et al., 2020). New studies have confirmed that 75 mg/kg caffeine per day, which is equivalent to 6 mg/kg per day in humans, could significantly improve liver lipid deposition, glucose metabolism, inflammation, and fibrosis in a mouse model of NASH induced by a high-trans fatty acid/high-carbohydrate diet, with the mechanism thought to be related to the improvement of abnormal gene expression in NASH by caffeine (Konstantinou et al., 2014). However, numerous studies have shown conflicting results on the effects of caffeine on humans and animals. Dungubat et al. used C57BL/6J mice fed a choline-deficient, L-amino acid-defined, high-fat diet (CDAHFD) as an animal model of NASH. Seven-week-old male C57BL/6J received 0.05% (w/w) caffeine and CDAHFD supplemented with 0.1% (w/w) caffeine and chlorogenic acid (CGA) for 7 weeks, and the results showed that caffeine and CGA significantly worsened the markers of liver cell injury, inflammation, and/or steatosis in NASH lesions in mice (Dungubat et al., 2020). Differences in experimental conditions may account for the conflicting results of studies on the effects of caffeine and CGA on NAFLD/NASH. Moreover, in humans, Birerdinc et al. (2012) reported a protective effect of caffeine on patients with NAFLD, but Shen et al. (2016) reported that total caffeine intake was not associated with the prevalence of hepatic fibrosis in NAFLD. Therefore, the use of caffeine in the prevention and treatment of NAFLD still needs further research.

However, another researcher investigated the effects of caffeine and green coffee extract (GCE) on hepatic lipids in lean female rats with steatosis (Velázquez et al., 2020). Female SD rats were fed a standard diet or a cocoa butter-based HFD plus 10% liquid fructose for 3 months, which induced hepatic steatosis without obesity, inflammation, endoplasmic reticulum stress, or hepatic insulin resistance. In the third month, the HFD was supplemented with caffeine (5 mg/kg/day) or GCE. However, the experimental results indicated that neither caffeine nor GCE alleviated hepatic steatosis, but the GCE-treated rats showed lower hepatic triglyceride levels compared with the caffeine group. The experimental results show that a low dose of caffeine did not reduce hepatic steatosis in lean female rats, but the same dose provided as a GCE reduced liver triglyceride levels. Thus, a moderate dose of caffeine, equivalent to 1 cup of coffee a day in humans, did not alleviate liver lipid deposition in a model of diet-induced hepatic steatosis without obesity and inflammation. Differences in experimental conditions may account for the conflicting results of studies on the effects of caffeine on NAFLD/NASH. Various NAFLD/NASH animal models have been used in various studies, mainly including nutritional models, such as high fat, high cholesterol, and high fructose diet, and genetic models such as KK-AY mice (Figure 2) (Birerdinc et al., 2012; Velázquez et al., 2020). Additionally, various caffeine administration methods are used in experimental studies, including *via* drinking water, diet, and gavage, and the doses vary across studies. Therefore, various factors lead to contradictory experimental results across different experiments.

### 2.4 Caffeine in cirrhosis

Cirrhosis is a common clinical chronic progressive liver disease, which is caused by one or more etiologies of chronic or repeated action of diffuse liver damage. The histopathology of cirrhosis shows extensive necrosis of hepatocytes, nodular regeneration of residual hepatocytes, connective tissue hyperplasia, and fibrous septum formation, resulting in the destruction of the liver lobule structure and formation of pseudolobules (Feld et al., 2015). The liver gradually becomes deformed and hardened before ultimately developing into cirrhosis. Earlier studies have found that caffeine clearance in cirrhosis can be used to measure liver metabolic capacity (Lewis and Rector, 1992; Tangkijvanich et al., 1999). For example, the 13C-caffeine breath test is a non-invasive, quantitative test of liver function, which can reliably differentiate patients with decompensated cirrhosis from non-cirrhotic patients with chronic liver diseases (Xin et al., 2021). Additionally, previous studies have assessed the effect of caffeinated beverage consumption on the risk of symptomatic liver cirrhosis (Corrao et al., 2001). The results demonstrated a significant reduction in the risk of liver cirrhosis with increased coffee intake. The liver cirrhosis odds ratios (ORs) decreased from 1.0 (reference category: lifetime abstainers from coffee) to 0.47 (95% confidence interval: 0.20, 1.10), 0.23 (0.10, 0.53), 0.21 (0.06, 0.74), and 0.16 (0.05, 0.50) in individuals who drank 1, 2, 3, and ≥4 cups of coffee, respectively. Caffeine (250 mg) is also commonly administered orally to investigate the effect of cirrhosis on the disposition of the liver and the elimination of drugs from it (Desmond et al., 1980). These findings suggest that coffee, but not other caffeinated beverages, inhibits the onset of alcoholic and nonalcoholic liver cirrhosis (Corrao et al., 2001).

The anti-inflammatory effect of caffeine varies with the treatment protocols and doses (Feld et al., 2015; de Alcântara Almeida et al., 2021). Bile duct ligation (BDL) in rats is a common animal model of liver cirrhosis, and previous studies have demonstrated that BDL rats have significantly higher portal pressure, elevated total bilirubin (TB), AST, and ALT compared to sham-operated rats, indicating the typical presentation of liver cirrhosis (Chang et al., 2019). Researchers have found that treatment with high doses of caffeine (50 mg/kg/day) for 2 weeks eased liver fibrosis, alleviated intrahepatic angiogenesis, and reduced portal pressure in rats with BDL-induced cirrhosis without adversely affecting systemic hemodynamics, supporting the benefit of using caffeine treatment in patients with cirrhosis (Chang et al., 2019). The underlying mechanisms might be related to inhibiting angiogenesis and inflammation. However, the plasma levels of ALT, AST, and TB were not influenced by 50 mg/kg/day dose of caffeine treatment in common BDL rats; therefore, the specific mechanisms require further study. It has been reported that 250 mg of caffeine administered orally can prolong the elimination half-life by approximately 1 h in patients with cirrhosis compared to healthy controls, but the result did not reach statistical significance (Desmond et al., 1980). Therefore, the adverse effects of prolonged plasma clearance should be considered prior to long-term oral administration of coffee in patients with cirrhosis (Lewis and Rector, 1992).

In the early stage of liver cirrhosis, there are no obvious symptoms due to the strong liver compensatory function, while in the later stage, liver function damage and portal hypertension are the main manifestations, and multiple systems are involved. In the late stage, complications, such as upper gastrointestinal bleeding, hepatic encephalopathy, secondary infection, hypersplenism, ascites, and cancer are common (Hsu et al., 2015). Oral caffeine at 50 mg/kg/day alleviates hemodynamic derangements and portal hypertension in cirrhotic rats induced by BDL compared to vehicle (sham rats). Additionally, 28 days after administration, caffeine increased systemic vascular resistance and reduced superior mesenteric artery flow, mesenteric vascular density, portosystemic shunting, intrahepatic angiogenesis, and fibrosis, without affecting liver and renal biochemistry (Hsu et al., 2015). Further research found that the selective adenosine A1 agonist N6-cyclopentyladenosine (CPA) (1 mg/kg/day, i.p.) or A2A agonist GCS21680 (0.5 mg/kg/day, i.p.) could reverse these treatment effects. Researchers then used oral gavage administration of TAA (200 mg/kg, thrice-weekly for 8 weeks)-induced cirrhotic rats, and the results demonstrated that caffeine downregulated endothelial NO synthase, vascular endothelial growth factor (VEGF), phospho-VEGFR2, and phospho-Akt mesenteric protein expression compared to vehicle (distilled water). Further *in vivo* studies have also found that caffeine inhibited the activation of hepatic stellate and sinusoidal endothelial cells, which was reversed by CPA (1 mg/kg/day, i.p.) and GCS21680 (0.5 mg/kg/day, i.p.) (Figure 3) (Hsu et al., 2015). However, caffeine did not modify the vascular response to vasoconstrictors in splanchnic, hepatic, and collateral vascular beds in this study, although the specific reasons for this remain to be determined (Hsu et al., 2015).

### 2.5 Caffeine in (autoimmune) hepatitis

Hepatitis is a general term for liver inflammation, which usually refers to liver cells destroyed by various pathogenic factors, such as viruses, bacteria, parasites, chemicals, poisons, drugs, alcohol, and autoimmune factors. The function of the liver is damaged, causing a series of uncomfortable symptoms and abnormal liver function indicators (Jaruvongvanich et al., 2017; Wijarnpreecha et al., 2017). A case control study, which employed 234 hepatitis B virus (HBV) chronic carriers (109 cases and 125 controls) investigated whether moderate coffee consumption reduces the risk of HCC in hepatitis B chronic carriers. The results indicated that coffee consumption significantly reduced the risk of HCC by almost half [OR: 0.54, 95% confidence interval (CI): 0.30–0.97] in HBV chronic carriers, with the risk to moderate drinkers reduced by almost 60% (OR: 0.41, 95% CI: 0.19–0.89) compared to those with no coffee-drinking habit (Leung et al., 2011). Caffeine inhibits hepatitis C virus (HCV) replication *in vitro* and has been shown to dose-dependently inhibit HCV replication at non-cytotoxic concentrations, with an IC50 value of 0.7263 mM after 48 h of incubation. These results implicate caffeine as a novel agent for anti-HCV therapies due to its efficient inhibition of HCV replication at non-toxic concentrations (Batista et al., 2015). Additionally, previous studies have demonstrated that caffeine intake is significantly associated with decreased odds of advanced hepatic fibrosis in patients with chronic hepatitis C. Future prospective studies should focus on assessing the optimal dose and preparation of caffeinated beverages for the prevention of hepatitis (Jaruvongvanich et al., 2017; Wijarnpreecha et al., 2017).

Autoimmune hepatitis (AIH) is one of the three major autoimmune liver diseases, in addition to biliary cirrhosis and primary sclerosing cholangitis. AIH is a chronic inflammation of the liver of unknown etiology and presents with hyperimmunoglobulinemia and/or circulating autoantibodies (Lammert et al., 2022). The basic pathology of AIH is detrital necrosis in the perilobular area of the liver, which may also be accompanied by bridging necrosis with marked infiltration of lymphocytes and monocytes. AIH is more common in females, with a male to female ratio of approximately 1:4. AIH can occur at any age and is often accompanied by extrahepatic autoimmune disease, which can be effectively treated with immunosuppressive therapy (Lammert et al., 2022). A recent study found that patients with AIH reported lower lifetime coffee consumption. Researchers investigated the lifetime coffee consumption of 358 patients with AIH (cases) and 564 volunteers (controls), as well as its associations with age, sex, education, smoking status, BMI, and daily activity. The results showed that 24.6% of patients with AIH never drank coffee compared to 15.7% of controls (*p* < 0.001). Additionally, only 65.6% were current drinkers compared to 77% of controls (*p* < 0.001). Besides, patients with AIH consumed fewer lifetime cups of coffee per month (45 vs. 47 for controls, *p* < 0.001) and spent a lower percentage of their lives drinking coffee (62.5% vs. 69.1% for controls, *p* < 0.001). Furthermore, the relationship between coffee consumption and the risk of AIH development was dose-dependent (Figure 3). However, the major limitations of this study include the memory bias of the patients with AIH and the low population of patients. Future studies should consider the association between coffee intake and outcomes related to non-transplanted AIH livers (Lammert et al., 2022).

### 2.6 Caffeine in HCC

HCC is a primary liver cancer with a high mortality rate and represents the most common malignancy worldwide, especially in Asia, Africa, and southern Europe (Johnson et al., 2011; Kawano et al., 2012). Viral hepatitis (hepatitis B or C), cirrhosis, diabetes mellitus, and chronic alcoholism are the most common causes of HCC (Johnson et al., 2022; Salem et al., 2022). Tumor size and stage are the two key factors affecting the treatment and prognosis of HCC, and the prognosis of advanced HCC is poor. Primary HCC is rare in the United States, with most cases being metastatic HCC (Higdon and Frei, 2006; Kennedy et al., 2017). Sorafenib was approved by the FDA in 2007 for treating unresectable HCC, while in 2017, regorafenib and nivolumab were approved for HCC. Lenvatinib was approved in 2018 for patients with unresectable HCC. All four of these drugs have drug resistance and toxicity, and their therapeutic effects remain incompletely satisfactory. According to recent reports, 60% of the anticancer medications in current use have been obtained from natural sources (Rawat et al., 2018). Dietary phytochemical caffeine has been found to be useful for treating HCC and other diseases (Fujimaki et al., 2012; Estari et al., 2021). As early as 1989, studies reported on the use of caffeine-potentiated chemotherapy (Kawano et al., 2012). Recent cellular assays, animal experiments, and clinical trials have shown that caffeine has a protective effect on the liver and reduces the risk of HCC. Caffeine may be developed as a drug to prevent and treat HCC in the future (Figure 3) (Okano et al., 2011; Wang et al., 2019).

#### 2.6.1 Cellular assays

Previous studies have found that caffeine inhibits DNA repair and can increase the antitumor effect of cisplatin (Chae et al., 2012; Kawano et al., 2012). Researchers have demonstrated that caffeine (0.5 mM) increased the antitumor effect of cisplatin (0.4, 0.6, 1.2, 0.9 μg/ml) on the proliferation of various HCC cells (Li-7, HLF, HuH-7, HepG2). However, caffeine treatment alone did not increase cell apoptosis in the four HCC cell lines (Chae et al., 2012; Kawano et al., 2012). Currently, the idea that caffeine reduces the risk of HCC is controversial (Wang et al., 2011). Caffeine is a well-known radiosensitizer, which eliminates radiation-induced G2 block and promotes cancer cell apoptosis. Caffeine can increase the radiosensitivity of McA-RH7777 rat hepatocellular cancer cells but had no effect on BRL3A rat normal liver cells and irradiated normal liver tissues *in vivo*. However, caffeine has been shown to enhance the radiosensitivity of ortho topically transplanted human HCC in a nude mouse model, suggesting that it may be effective intreating liver cancer, although the dosage and method require further study (Wang et al., 2011). Chae et al. demonstrated that paclitaxel combined with caffeine is effective in the reversal of paclitaxel resistance through the inhibition of the DNA damage checkpoint *in vitro* (Chae et al., 2012). The results of these cell assays support the use of caffeine as an adjunctive therapy for HCC (Wang et al., 2011; Chae et al., 2012). Another cellular assay found that the non-selective AR antagonist caffeine and its analog CGS 15943 inhibited human HCC cell growth and the proliferation of four HCC cell lines (HLF and SK-Hep-1 cell lines, HepG2 and PLC-PRF-5 cells) by targeting the phosphoinositide 3-kinase/Akt pathway; however, CGS 15943 only slightly induced apoptosis in these cell lines, suggesting that caffeine has a chemopreventive effect against HCC (Edling et al., 2014). Moreover, an *in vitro* study showed that a low concentration of caffeine inhibits the progression of HCC *via* the Akt signaling pathway (Dong et al., 2015), while another study showed that caffeine inhibited the proliferation of liver cancer cells and activated the MEK/ERK/EGFR signaling pathway (Okano et al., 2008).

#### 2.6.2 Animal experiments

Currently, three chemical toxins, namely CCl_4_, TAA, and DEN, are commonly used to induce HCC in rodents, while there are more than 1,000 chemical compounds in coffee (Bravi et al., 2013; Rawat et al., 2018; Zhang et al., 2021). The anticancer effect of coffee may be the result of the combined action of several chemical components such as caffeine, trigonelline (TRI), and CGA (Kennedy et al., 2017; Romualdo et al., 2020). Indeed, researchers have demonstrated that a combination of CAF+TRI+CGA (50, 25, and 25 mg/kg) administered intragastrically for 10 weeks reduced the incidence, number, and proliferation (Ki-67) of hepatocellular preneoplastic foci while enhancing apoptosis (cleaved caspase-3) in the adjacent parenchyma in HCC rats induced by DEN/CCl_4_ (Romualdo et al., 2020). The mechanism by which the three component combination attenuates fibrosis-associated hepatocarcinogenesis may be related to reduced DNA expression. Different types of coffee may produce different results. It has also been shown that intake of conventional coffee, decaffeinated coffee, and 0.1% caffeine for 8 weeks significantly reduced serum ALT (*p* < 0.001), oxidized glutathione (*p* < 0.05), fibrosis/inflammation scores (*p* < 0.001), collagen volume fraction (*p* < 0.01), and TGF-β1 protein expression (*p* ≤ 0.001) in the livers from TAA (200 mg/kg b.w., i.p.)-treated Wistar rats (Amer et al., 2017).

#### 2.6.3 Clinical trials

Many epidemiological surveys and clinical studies have found that moderate coffee consumption reduces the risk of HCC, with similar results found in the North American population, the European population, the Japanese population, the Singaporean population, and the Hong Kong population (Bamia et al., 2015; Petrick et al., 2015; Di Maso et al., 2021). However, no such study has been conducted in the Chinese mainland population, which may be related to the fact that the Chinese population is not used to drinking coffee but prefers drinking tea (Johnson et al., 2011; Di Maso et al., 2021). From 1993 to 1998, the Singapore Chinese Health Study enrolled a prospective cohort of 63,257 middle-aged and older Chinese men and women with a relatively high-risk for HCC. The researchers found that 362 of the cohort had developed HCC by the end of 31 December 2006 and that individuals who consumed ≥3 cups of coffee per day experienced a statistically significant reduction in the risk of HCC [44%; hazard ratio (HR): 0.56, 95% CI: 0.31–1.00; *p* = 0.049] after adjustment for potential confounders and tea consumption compared to non-drinkers of coffee (Johnson et al., 2011). A recently published systematic review and dose-response meta-analysis enrolled 2,272,642 participants and 2,905 cases to explore the effects of coffee, including caffeinated and decaffeinated coffee, on the risk of HCC and found that an extra two cups of coffee per day was associated with a 35% reduction in the risk of HCC [relative risk (RR): 0.65, 95% CI: 0.59–0.72], while an extra two cups of caffeinated and decaffeinated coffee (2 and 3 cohort studies, respectively) was associated with reductions of 27% (RR: 0.73, 95% CI: 0.63–0.85) and 14% (RR: 0.86, 95% CI: 0.74–1.00) in the risk of HCC, respectively (Kennedy et al., 2017). The researchers believe that the mechanism of action of caffeine against HCC might be related to the inhibition of HCC cell proliferation and the alleviation of oxidative DNA damage, although opposing results have also been published (Kennedy et al., 2017). Indeed, another *in vitro* study found that caffeine alone at tested concentrations was not cytotoxic and did not induce DNA damage in HepG2 cells (Leão et al., 2021). Coffee consumption may have a protective effect on chronic liver disease, which needs to be confirmed in larger populations (Johnson et al., 2011). A Hong Kong, China, case-control study enrolled 234 chronic carriers of hepatitis B (109 vs. 125 controls) and found that moderate coffee consumption was associated with a reduced risk of HCC in chronic carriers of hepatitis B; however, the study was limited by a small sample size (Leung et al., 2011). Recent studies have demonstrated that the methylxanthine derivative caffeine upregulated peroxisome proliferator-activated receptor gamma (PPARγ) expression in hepatocytes of Caucasian patients with ongoing hepatic fibrogenesis and Chinese patients with fully developed HCC, suggesting that the effects of caffeine vary among ethnic groups; thus, further studies are necessary to fully understand the underlying mechanisms (Gressner et al., 2009).

The number of large clinical trials linking coffee consumption to a reduced risk of chronic liver disease has increased recently (Kennedy et al., 2017). A study published in 2015 recruited a total of 1,212,893 subjects [HCC, *n* = 860; intrahepatic cholangiocarcinoma (ICC), *n* = 260] and found that higher coffee consumption (>3 cups/day) was associated with lower risk of HCC compared to non-drinkers (HR: 0.73, 95% CI: 0.53–0.99; *p* < 0.0001). Additionally, coffee with or without caffeine and sex may have influenced the results, but there was no relationship between coffee consumption and ICC. Moreover, the large clinical trials did not consider ethnic differences in people from different regions and how the coffee was prepared (Petrick et al., 2015). Another multi-center, prospective cohort study identified 201 HCC cases among 486,799 men/women, after a median follow-up of 11 years and calculated the adjusted HR for HCC incidence in relation to quintiles/categories of coffee/tea intakes. The results showed that the coffee consumers in the highest quintile compared to the lowest quintile had a 72% reduced risk of HCC (HR: 0.28; 95% CI: 0.16–0.50, P-trend<0.001) (Bamia et al., 2015). Mechanistic studies could clarify whether these significant associations can be attributed to caffeine or other components in these drinks (Bamia et al., 2015). Two reviews published in 2021 analyzed and summarized the Caffeinated Coffee Consumption and Health Outcomes in the US Population and found that caffeinated coffee consumption decreased the incidence/mortality of cardiovascular disease, as well as the incidence of type 2 diabetes and HCC, with a recommended dose of 3–4 cups per day (120 ml/cup) (Di Maso et al., 2021; Kolb et al., 2021). Interestingly, coffee consumption has also been shown to reduce HCC recurrence after orthotopic liver transplantation, in that those with postoperative coffee intake ≥3 cups/day had a longer overall survival than those who consumed less or no coffee. An *in vitro* study showed that the antagonist activity of caffeine has adenosine A2AR-mediated growth-promoting effects on HCC cells associated with the MAPK and NF-kappa B pathways (Wiltberger et al., 2019). The specific mechanism by which coffee consumption reduces the risk of HCC still needs further study. In conclusion, recent cellular assays, animal experiments, and epidemiological studies have found that oral coffee consumption reduces the risk of HCC.

### 2.7 Toxicity or adverse effects of caffeine

**FIGURE 4:**
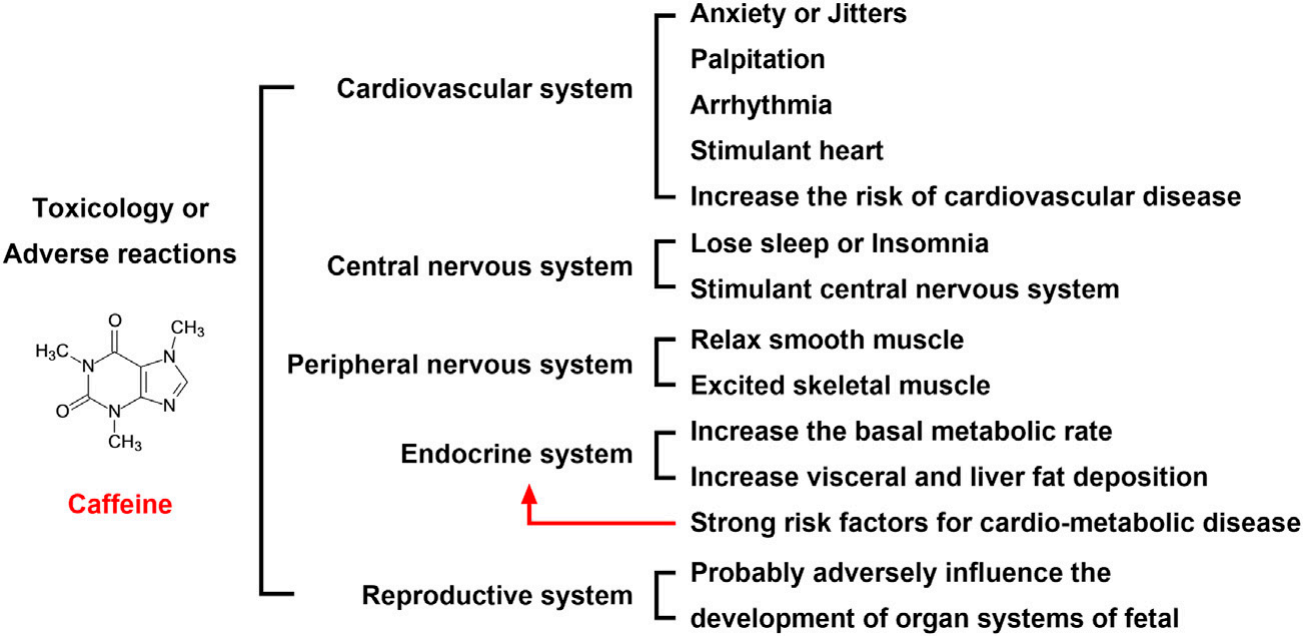
Toxicology or adverse reactions of caffeine. The adverse reactions of caffeine on the cardiovascular, nervous, and reproductive systems are serious. Although most of the adverse reactions disappear after discontinuing oral administration, the impact on the fetus is more serious, which requires expecting mothers to be vigilant. Therefore, an oral dose of coffee is not recommended for women who are trying to become pregnant or who are pregnant.

Maternal caffeine intake during pregnancy is associated with an increased risk of childhood obesity. Studies in adults suggest that caffeine intake also directly affects the visceral and liver fat deposition, which are strong risk factors for cardio-metabolic disease (Voerman et al., 2020) (Figure 4). A recent population-based prospective cohort study recruited 4,770 pregnant women, and of the 4,770 women included, 2,780 (58.3%), 1,583 (33.2%), 329 (6.9%), and 78 (1.6%) consumed <2 units, 2–3.9 units, 4–5.9 units, and ≥6 units of caffeine per day, respectively, during pregnancy. The results showed that high maternal caffeine intake during pregnancy was associated with higher childhood general body fat mass, abdominal fat mass, and liver fat fraction at the age of 10 years, while differential fat accumulation in these depots may increase susceptibility to cardio-metabolic disease in later life (Voerman et al., 2020). However, the mechanisms underlying these findings are poorly understood.

According to the World Health Organization (WHO), epidemiological evidence indicates that in pregnant women, a caffeine intake of 300 mg/day (5 mg/kg × day) is associated with an increased risk of intrauterine growth retardation (IUGR) (He et al., 2019). Previous studies have demonstrated that prenatal caffeine exposure (PCE) induces IUGR and high susceptibility to NAFLD in offspring rats, with the underlying mechanisms considered to be associated with fetal overexposure to maternal glucocorticoids (He et al., 2019). Further research found that PCE structurally and functionally inhibited the fetal liver and induced catch-up growth after birth in rat offspring with IUGR, and the mechanisms underlying these phenomena were associated with the inhibition of GR/C/EBPα/IGF1R signaling by intrauterine maternal glucocorticoid overexposure. The effects of caffeine exposure during pregnancy on the increased risk of liver disease in offspring remain controversial and require further study. Another study showed that PCE increased the serum total cholesterol levels in adult offspring rats *via* impacting histone acetylation and cholesterol synthesis-related gene expression *via* regulation of the A2AR/cAMP/PKA pathway and SIRT1 expression at the cellular level (Hu et al., 2019). Therefore, oral caffeine (including caffeinated, decaffeinated, or a combination of both) consumption at any dose is not recommended for women who are trying to become pregnant or who are already pregnant. Interestingly, maternal caffeine intake, but not paternal caffeine intake, has been found to be associated with childhood obesity (Voerman et al., 2020).

## 3 Discussion

The present study examined the beneficial use of caffeine in different liver diseases and its toxity. The pharmacokinetics of caffeine in human body and possible adverse effects of coffee/caffeine consumption have been investigated in great details. This systematic review of studies on the effects of coffee on ALD, viral (autoimmune) hepatitis, NAFLD, cirrhosis and HCC was performed to examine the association of coffee consumption with various chronic liver diseases. Many questions however remain unanswered before we conclude that coffee can take care of our livers. Amount of coffee, and type of coffee no doubt would be important but equally important might be the race of the population being studied for the beneficial effects of coffee. Additional animal and cell culture studies have also further elucidated the biochemical basis by which coffee may inhibit adenosine and AR binding in patients with various liver diseases, and the mechanism involves multiple signaling pathways. Besides, at present, the experimental results on the effects of coffee exposure during pregnancy on the fetus are still controversial and need further study. There is still a long way to go before all the above problems can be solved.

Numerous preclinical studies and epidemiological investigations have found that oral caffeine has preventive and therapeutic effects on various liver diseases. However, the current research on caffeine dosage remains controversial. In humans with a moderate to high caffeine intake, daily doses are approximately 10–20 mg/kg. It is estimated that an adult human (mean weight, 70 kg) with moderate coffee intake consumes approximately 400 mg of caffeine per day, which is equivalent to 6 mg/kg per day or three cups of coffee. The average green tea drinker could be defined as a 60 kg man who consumes 10 g of green tea in four cups every day. In the future, caffeine may be developed as a drug to prevent and treat liver diseases. Future research should focus on identifying the explicit dose and mechanisms.
